# Cerebrovascular Manifestations in a Family with a Pathogenic *COL4A5* Variant

**DOI:** 10.3390/jcm15197350

**Published:** 2026-09-22

**Authors:** Hagit Toledano-Alhadef, Aviva Fattal Valevski, Moran Hausman-Kedem, Shlomi Constantini, Shelly Shiran, Anat Oron, Keren Yosovich, Tally Lerman-Sagie, Marina Michelson

**Affiliations:** 1 Gilbert’s Israeli and International Neurofibromatosis Center, Tel Aviv-Yafo 6423906, Israel; 2 Pediatric Neurology Institute and Child Development Center, Dana-Dwek Children’s Hospital, Tel Aviv Sourasky Medical Center, Tel Aviv-Yafo 6423906, Israel; 3 The Gray Faculty of Medical and Health Sciences, Tel Aviv University, Tel Aviv-Yafo 6997801, Israel; 4 Department of Pediatric Neurosurgery, Tel Aviv Sourasky Medical Center, Tel Aviv-Yafo 6423906, Israel; 5 Pediatric Radiology Unit, Dana-Dwek Children’s Hospital, Tel Aviv Sourasky Medical Center, Tel Aviv-Yafo 6423906, Israel; 6 Medical Division, Maccabi Healthcare Services, Tel Aviv-Yafo 6816330, Israel; mashakerman@gmail.com; 7 Institute of Medical Genetics, Wolfson Medical Center, Holon 5822012, Israel; 8 Multidisciplinary Fetal Neurology Center, Fetal Brain Research Center, Wolfson Medical Center, Holon 5822012, Israel; 9 Adelson School of Medicine, Ariel University, Ariel 40700, Israel

**Keywords:** *COL4A5*, vasculopathy, intracranial hemorrhage, cerebrovascular disease, founder variant, segmental neurofibromatosis

## Abstract

**Background/Objectives**: Pathogenic variants in collagen type 4 α5 chain (*COL4A5)*, responsible for X-linked Alport syndrome, are classically associated with progressive kidney disease, sensorineural hearing impairment, and ocular abnormalities. Cerebrovascular involvement is not considered part of the COL4A5-related phenotype, although rare recent reports suggest it may occur. We describe a family with cerebrovascular events across three generations, segregating in a clear X-linked pattern, and evaluate whether these events could be linked to a pathogenic *COL4A5* variant identified in the proband. **Methods**: We report the clinical, radiological, and genetic findings in a 16-year-old heterozygous female proband, interpreted in the context of her family’s cerebrovascular history. Exome sequencing, chromosomal microarray, and familial segregation analysis were performed. **Results**: The proband presented with severe migraine, cerebellar microhemorrhage, and poor visualization of the left distal posterior cerebral artery (PCA) branches. Exome sequencing detected the heterozygous *COL4A5* pathogenic variant c.1871G>A; p.(Gly624Asp), inherited from her hemizygous father, who has Alport syndrome. The proband’s sister, an obligate carrier of the same variant with a history of migraine, was found to have a small brain aneurysm. In the paternal line, the great-grandmother died of a cerebrovascular event in her early 40 s, the great-aunt had a stroke at around 40, and her son died of a cerebrovascular event at 35; the obligate-carrier grandmother had chronic kidney disease and migraine. **Conclusions**: This *COL4A5* variant is usually associated with late-onset Alport syndrome and variable renal involvement, even in obligate carriers, and has not previously been linked to cerebrovascular disease. In the present family, the X-linked clustering of cerebrovascular events raises the possibility that COL4A5 may contribute to cerebrovascular fragility, with an effect that may extend beyond the kidney. These findings suggest that COL4A5 may be relevant in selected families with unexplained cerebrovascular disease, even when renal manifestations are mild or absent.

## 1. Introduction

Type IV collagen is an essential structural component of vascular basement membranes. Pathogenic variants in the genes encoding type IV collagen are increasingly recognized as important contributors to cerebrovascular disease [1]. *COL4A1* and *COL4A2* variants are well-established causes of cerebral small vessel disease, including porencephaly, intracerebral hemorrhage, and lacunar infarcts, attributed to fragility of the cerebral vessel wall [2,3,4,5]. Type IV collagens constitute the structural network of basement membranes through specific heterotrimeric assemblies. Within this network, *COL4A5* encodes the α5 chain of type IV collagen, core component of the glomerular basement membrane. Pathogenic variants in *COL4A5* cause X-linked Alport syndrome (XLAS), characterized by hematuria, progressive renal failure, sensorineural hearing loss, and ocular abnormalities [6,7]. Given the widespread distribution of type IV collagen in vascular basement membranes, involvement of the cerebral vasculature would not be unexpected. Recent reports describing intracranial arterial stenosis, aneurysms, and fibromuscular dysplasia in individuals with *COL4A5* variants raise the possibility that the underlying basement membrane defect extends beyond the kidney, supporting a potential link between *COL4A5* and cerebrovascular disease [8,9,10,11,12]. We report a 16-year-old female patient with migraine in whom brain magnetic resonance angiography (MRA) revealed a cerebellar microhemorrhage and poor visualization of the left distal posterior cerebral artery (PCA) branches. Exome sequencing identified a heterozygous *COL4A5* missense pathogenic variant (c.1871G>A; p.Gly624Asp), inherited from her father, a known patient with Alport syndrome with persistent hematuria, progressive renal dysfunction, and mild sensorineural hearing loss.

Three relatives in the paternal lineage had non-traumatic intracranial hemorrhages, in a pattern compatible with X-linked inheritance. The proband also had clinical features of segmental neurofibromatosis type 1, a mosaic disorder in which the causative NF1 variant may not be detectable in peripheral blood. Cerebrovascular involvement in individuals with *COL4A5* variants has been reported only rarely, with two cases described to date [13,14,15].

We report the clinical, radiological, and genetic findings of a family carrying the pathogenic *COL4A5* c.1871G>A (p.Gly624Asp) variant and discuss its possible relevance to the observed cerebrovascular manifestations.

## 2. Clinical Presentation

A previously healthy 16-year-old adolescent female of Serbian–Iraqi Jewish ancestry, presented with her first acute episode of transient focal neurological symptoms. The episode began with gradually progressive paresthesia involving the right hand, which subsequently spread to the forearm and upper arm. The sensory symptoms were followed by numbness and weakness of the right upper limb. There was no associated facial involvement, lower extremity weakness, dysarthria, aphasia, visual disturbance, impaired consciousness, abnormal movements, or sphincter dysfunction. She subsequently developed a unilateral throbbing headache unaccompanied by other symptoms. The focal neurological deficits resolved completely within ten minutes, whereas the headache persisted for several hours and improved gradually. Notably, the episode occurred in close temporal proximity to the onset of menstruation. Based on the temporal evolution of the neurological deficits followed by a typical migrainous headache, the episode was considered consistent with a first presentation of migraine with aura.

The patient had no previous history of migraine, seizures, cerebrovascular disease, or other neurological disorders. She was not taking any regular medications, and neurological examination after symptom resolution was normal, with no residual focal deficits. Her past medical history was unremarkable. She had an uneventful perinatal course, with no reported antenatal, perinatal, or neonatal complications. Growth and developmental milestones were appropriate for age and were achieved within the expected timeframes. There was no history of developmental delay or other significant medical concerns during childhood. Thereafter, her medical history was notable for the development of multiple irregularly shaped café-au-lait macules predominantly distributed along the right side of the body, with the greatest concentration over the right shoulder and abdominal region. The lesions gradually increased in size and became more prominent with age. Right axillary freckling was also observed. Despite these cutaneous findings, there were no other clinical features suggestive of neurofibromatosis type 1 (NF1) and the patient did not meet the clinical criteria for NF1 nor segmental NF1. In particular, ophthalmological assessment revealed no evidence of Lisch nodules or other characteristic ocular abnormalities, including choroidal lesions. There were also no clinical features of NF1-associated osseous abnormalities. Neurological and developmental assessments were otherwise unremarkable, with no evidence of global neurodevelopmental impairment, although mild learning difficulties were reported. There was no family history of neurofibromatosis type 1 (NF1) or café-au-lait macules.

The patient’s father had persistent microhematuria and mild hearing impairment from his 40s, consistent with Alport syndrome. On the paternal side, several family members had experienced non-traumatic intracranial hemorrhages, including two relatives who died suddenly following catastrophic intracranial hemorrhage (Figure 1).

Brain magnetic resonance imaging (MRI) of the patient demonstrated a punctate susceptibility focus within the left cerebellar hemisphere, consistent with a cerebral microbleed (Figure 2).

Intracranial magnetic resonance angiography (MRA) showed poor visualization of the left distal PCA branches, raising the possibility of congenital hypoplasia or chronic arterial occlusion (Figure 3). Contrast-enhanced MRA of the abdomen demonstrated a focal short-segment stenosis of the proximal left renal artery, as well as additional stenotic changes involving the bilateral lobar renal artery bifurcations, suggestive of multifocal renovascular involvement.

The proband’s 24-year-old sister was previously healthy, with normal perinatal and developmental history and no known systemic disease. She was a university student and reported severe migraine, particularly during examination periods. Following the proband’s presentation and the striking paternal family history of early cerebrovascular events, she underwent clinical and genetic evaluation. She was found to carry the same pathogenic *COL4A5* c.1871G>A; p.(Gly624Asp) variant. Brain MRA demonstrated a small saccular aneurysm versus a prominent small branch artery infundibulum in the Anterior communicating artery (Acom) region (Figure 4A,B).

Serial urinalyses in the proband and her sister showed no microscopic hematuria, albuminuria, or proteinuria. Urine albumin/creatinine ratio was within the normal range. Serum creatinine and calculated eGFR were normal in both sisters. Blood pressure measurements were within normal limits. Renal ultrasound showed no abnormal findings.

Detailed records for the affected relatives were unavailable. The two relatives that had died years earlier, and the surviving relatives could not be re-evaluated. Consequently, the family history was based on reported fatal non-traumatic cerebrovascular events rather than direct clinical or neuroimaging documentation.

## 3. Materials and Methods

This retrospective three-generation family study included clinical, radiological, and molecular genetic evaluation of the proband and available family members. Genotyping was performed in the living family members, including the proband her sister and her parents. Clinical information regarding the deceased relatives was obtained through structured family interviews and available medical records.

Genomic DNA was extracted from peripheral blood. Whole-exome sequencing was performed using the Nextera DNA Flex Pre-Enrichment Library Prep kit together with the IDT xGen Exome Research Panel v2. Sequencing was carried out on a NovaSeq 6000 platform (Illumina, San Diego, CA, USA) using 100-bp paired-end reads. Sequence reads were aligned to the human reference genome (GRCh37/hg19). Bioinformatic analysis was performed using the Genoox platform, incorporating BWA (version 0.7.16) for sequence alignment and GATK HaplotypeCaller (version 3.7) together with FreeBayes (version 1.1.0) for variant calling. Variants were interpreted according to the American College of Medical Genetics and Genomics/Association for Molecular Pathology (ACMG/AMP) recommendations in the context of the patient’s clinical phenotype. Pathogenic and likely pathogenic variants were reported routinely, whereas variants of uncertain significance were reported only when considered clinically relevant to the presenting phenotype. Genes associated with hereditary vasculopathy were evaluated as part of the whole-exome analysis. Familial segregation of the identified COL4A5 variant was confirmed by direct Sanger sequencing (3500 Genetic Analyzer, Applied Biosystems, Waltham, MA, USA). SNP chromosomal microarray analysis was performed on DNA extracted from peripheral blood using the Illumina Gene by Gene GxG ComprehensiveArray v2.2 BeadChip (731,004 SNP loci; genome build 37).

## 4. Results

A pathogenic heterozygous variant in the X-linked *COL4A5* gene: *COL4A5* (NM_033380.3): c.1871G>A;p.Gly624Asp was detected. No pathogenic variants were identified in *NF1*, *SPRED1*, *COL4A1*, *COL4A2* and other genes known to be associated with vasculopathies.

Further segregation in the family revealed that the patient and her sister inherited this variant from the father, of Serbian ancestry, who was hemizygous. The mother did not carry the variant.

Chromosomal microarray of the proband showed a normal female karyotype; arr(1-22,X)x2 without evidence for deletions or insertions in known regions of genetic syndromes. Further evaluation, including renal ultrasound and audiometric testing and ophthalmology examinations, showed normal results.

## 5. Discussion

The present findings point to a possible contribution of the *COL4A5* variant to the cerebrovascular vulnerability observed in this family, although the evidence remains indirect. The proband, a 16-year-old heterozygous female carrying the c.1871G>A (p.Gly624Asp) variant, presented with an episode of migraine with aura and was found to have a cerebellar microhemorrhage and radiologic findings suggestive of an underlying arterial malformation at a young age. The 24-year-old previously healthy sister was identified through segregation studies as a carrier of the familial variant. Despite having no evidence of renal involvement and normal hearing, she reported a history of severe migraine episodes. Subsequent neuroimaging performed as part of her clinical evaluation revealed a small cerebral aneurysm.

This is particularly notable, as cerebrovascular presentations in *COL4A5*-related disease have so far been described almost exclusively in adults [8,9,10,11,12], as summarized in Table 1.

The published evidence linking *COL4A5* to cerebrovascular disease is scarce. Only two of the five reports include a confirmed *COL4A5* variant [9,12]. One study documented a woman with multiple intracranial artery stenoses, whose son carried the same variant hemizygously. Another report describes a woman with vertebral dissection and fibromuscular dysplasia, her asymptomatic mother and daughter carried the same *COL4A5* and *COL4A6* variants.

Additionally, three independent literature reports identified major cerebrovascular complications in patients with clinically diagnosed XLAS without molecular confirmation: a ruptured carotid artery aneurysm in a 14-year-old male, a basilar artery dissection in a female patient, and a vertebral artery dissection extending into the vertebrobasilar junction in a male patient [8,10,11]. Over the past two decades, the literature has remained limited to isolated case reports, with genetic confirmation in only two cases. The present pedigree provides further clinical and genetic observations. The pathogenic COL4A5 variant was identified in the 16-year-old proband and was confirmed by segregation analysis in her hemizygous father, who had clinically confirmed Alport syndrome, as well as in her 24-year-old sister. Although the father had no reported cerebrovascular disease, three further cerebrovascular events occurred along the same paternal lineage. These findings raise the possibility of an association between the pathogenic *COL4A5* variant and cerebrovascular disease. Genetic testing was unavailable for the other relatives. The interpretation therefore relies on clinical family history, and three points should be considered. First, the cerebrovascular events occurred along the paternal line, in a pattern compatible with X-linked transmission. Second, p.(Gly624Asp) is a recurrent founder allele rather than a private variant. Third, the variant is associated with thin glomerular basement membrane, indicating structural alteration of the collagen IV network despite its usually mild renal phenotype.

What most strongly influences our interpretation is the family history: the paternal grandmother had chronic kidney disease, and three additional paternal relatives experienced severe or fatal non-traumatic intracranial hemorrhage. This clustering, while not conclusive, may reflect more than chance and raises the possibility of a familial cerebrovascular susceptibility segregating with the X-linked *COL4A5* variant.

The absence of reported cerebrovascular disease in the father should be interpreted with caution. His renal and auditory findings are consistent with the expected phenotype of a hemizygous male with X-linked Alport syndrome due to *COL4A5*. His daughters are heterozygous carriers, and the absence of renal or auditory manifestations at their current ages is not unexpected, since these features in females may be mild, late-onset, or absent. The possible cerebrovascular phenotype may follow a different pattern. If *COL4A5* contributes to vascular fragility, this manifestation may not be strictly allele-dose-dependent, and may be influenced by incomplete penetrance, age, and additional modifying factors. This is consistent with the known variable expression of *COL4A5*-related disease within families. The *COL4A5* c.1871G>A, p.(Gly624Asp) variant is a well-characterized pathogenic variant and a known founder allele in Central and Eastern Europe. It is typically associated with a mild Alport phenotype, isolated microhematuria, late onset slowly progressive renal disease, and mild or progressive hearing impairment. Heterozygous females are frequently asymptomatic or minimally affected [16,17]. In a recent Central European cohort, it accounted for 16% of all genetic diagnoses of Alport syndrome, with a gnomAD frequency of 0.0087% [18]. Cerebrovascular risk for p.(Gly624Asp) cannot be assessed from the available published data. GnomAD provides allele-frequency data only, and the existing p.(Gly624Asp) Alport cohorts focus mainly on renal and auditory manifestations, without systematic reporting of stroke, aneurysm, or other cerebrovascular events.

As a recurrent founder allele, the variant is unlikely to represent a de novo finding in the father. Extrarenal manifestations, including hearing impairment and ocular involvement, have been reported less frequently among p.Gly624Asp carriers, although this difference did not reach statistical significance. Severe presentations have also been reported, indicating that the phenotype may be influenced by additional genetic factors [19]. COL4A5 is a component of basement membranes in the kidney, eye, and inner ear. Its expression has also been demonstrated in embryonic and adult pial fibroblasts and in zebrafish meningeal fibroblasts [20]. These findings provide biological context for considering a possible role outside the kidney, although direct evidence from human cerebral vascular tissue is currently lacking. The mechanism by which *COL4A5* variants might contribute to vascular pathology remains unclear. *COL4A1* and *COL4A2* are established monogenic causes of cerebral small-vessel disease, and their most characteristic pathogenic variants are missense glycine substitutions in the collagenous triple-helical domain that increase susceptibility to brain hemorrhage [1,21,22,23]. Egashira et al. [12], described a patient with Alport syndrome, moyamoya vessels, and cerebral aneurysms. They proposed that, because type IV collagen α5 is a component of the vascular basement membrane and contributes to smooth muscle stability, missense variants in *COL4A5* may weaken the smooth muscle-rich tunica media of cerebral arteries and thereby increase vascular vulnerability. To the best of our knowledge, cerebrovascular involvement has not previously been reported in association with p.Gly624Asp specifically.

A mild renal phenotype does not imply an intact collagen IV network. The *COL4A5* c.1871G>A variant preserves partial α5(IV) function rather than causing complete loss of function, allowing some α3α4α5(IV) network assembly with reduced stability. This variant is known to cause thinning of the glomerular basement membrane, indicating that the collagen IV network remains structurally altered [24]. Such a basement membrane abnormality may not be limited to the kidney and could similarly affect other basement membranes, including the vascular basement membrane. The proband also had segmental NF1. Although no germline NF1 variant was identified in blood, this does not exclude mosaic disease in a segmental presentation. To our knowledge, a direct link between NF1-related mechanisms and the collagen IV network has not been reported. In the present case, segmental NF1 is therefore better regarded as a possible comorbidity rather than a part of a primary mechanism.

Several limitations should also be acknowledged. Neuroimaging and renal data from the deceased relatives in the paternal line were not available. The historical cerebrovascular events in the paternal lineage were based on family history, without available genetic testing or neuroimaging documentation. Therefore, these events should be regarded as supportive family history rather than genotype-confirmed cases.

The absence of documented Alport manifestations therefore does not exclude *COL4A5*-related disease in these relatives. We also cannot exclude other rare factors contributing to cerebrovascular disease in this family. At the same time, the pedigree structure, the grandmother’s chronic kidney disease, the presence of the recurrent *COL4A5* p.(Gly624Asp) variant, and the cerebrovascular involvement in two sisters together keep *COL4A5* high among the possible explanations. High-resolution vessel-wall MRI was not available in the living carriers. Such imaging could help distinguish a true vessel-wall abnormality from a normal vascular variant, as shown in the genotype-confirmed case reported by Egashira et al. [12] This should be considered in future evaluation. Overall, the observations in this family should be interpreted as hypothesis-generating. They do not establish causality but support further evaluation of *COL4A5* in larger genotype-confirmed cohorts with systematic cerebrovascular phenotyping. We hypothesize that, in this family, the *COL4A5* c.1871G>A, p.(Gly624Asp) variant may have contributed to an underlying susceptibility to intracranial hemorrhage in addition to its role in the Alport spectrum phenotype. This association has not been consistently described in unselected p.(Gly624Asp) cohorts, but the clustering of three intracranial hemorrhages, two of them fatal, among paternal relatives supports this possibility.

## 6. Conclusions

This family study raises the possibility that the *COL4A5* c.1871G>A; p.(Gly624Asp) variant may contribute to cerebrovascular fragility beyond its established renal phenotype. *COL4A5* may also be considered in selected cases of unexplained familial cerebrovascular disease, even when renal or auditory manifestations are mild or absent. Larger genotype-confirmed cohorts are needed to clarify the strength of this association.

## Figures and Tables

**Figure 1:**
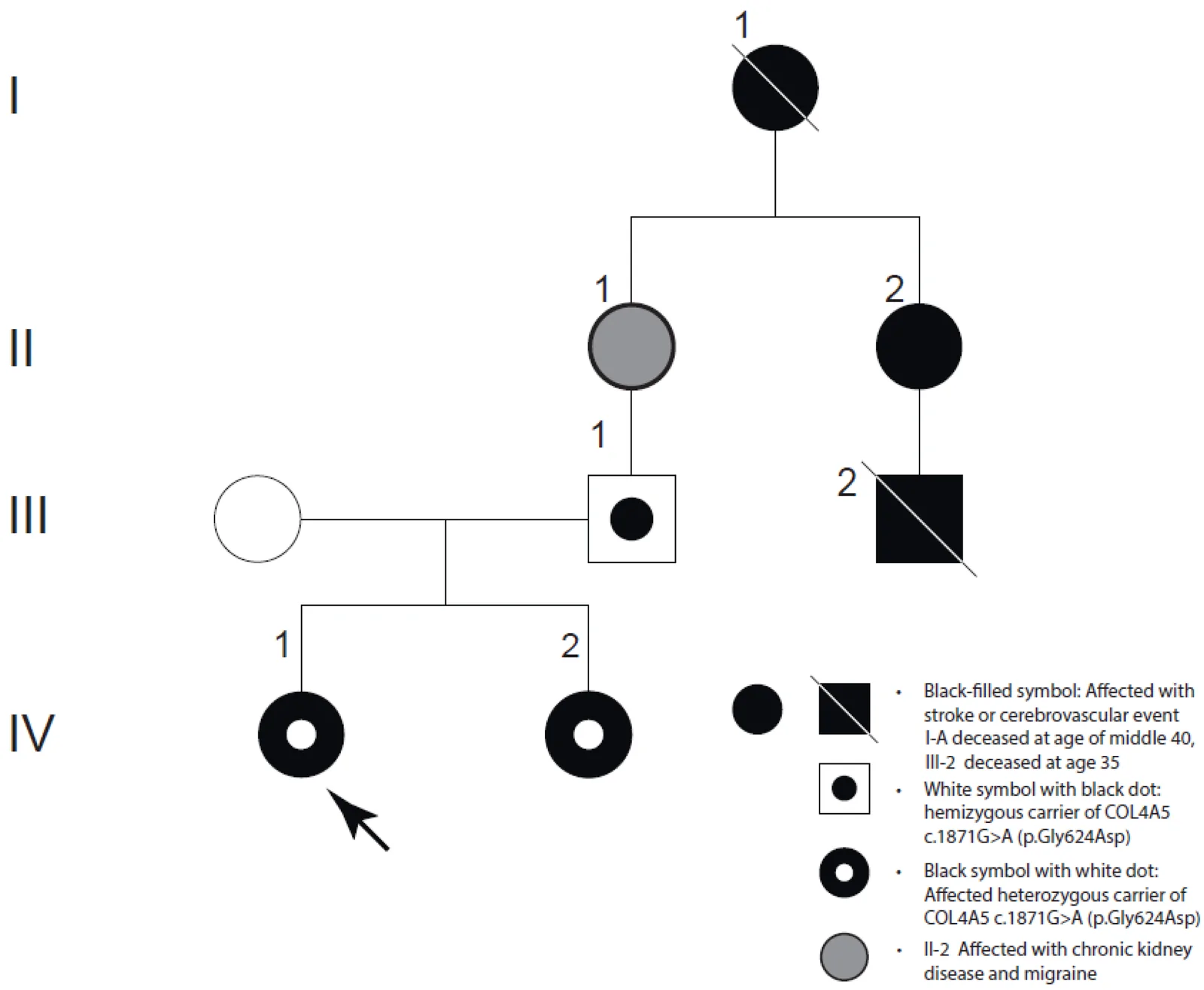
The proband presents with cerebellar hemorrhage and segmental neurofibromatosis. She is heterozygous for *COL4A5* NM_033380.3: c.1871G>A, p.(Gly624Asp). Her sister, an obligate and genetically confirmed heterozygous carrier, was evaluated because of migraine; brain MRA demonstrated cerebrovascular anomalies. The father is hemizygous for the same variant and has been diagnosed with Alport syndrome. The paternal great-grandmother, paternal great-aunt, and her son had early-onset cerebrovascular disease and stroke.

**Figure 2:**
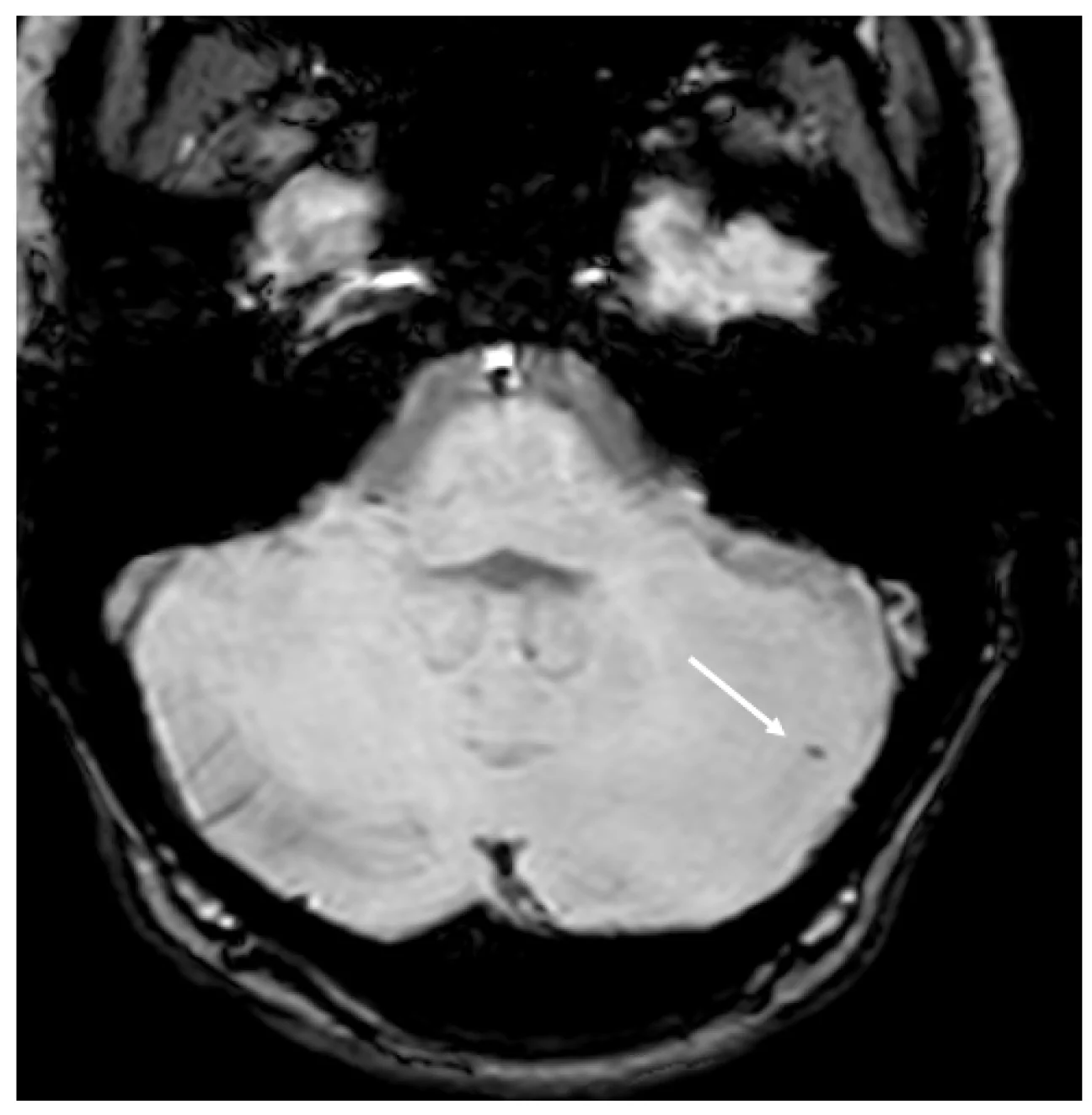
Axial susceptibility weighted image (SWI), from brain MRI study at presentation, demonstrates a small focus of increased susceptibility artifact in the left cerebellar hemisphere periphery (arrow), consistent with hemosiderin deposit, which may reflect a microbleed or small cavernoma.

**Figure 3:**
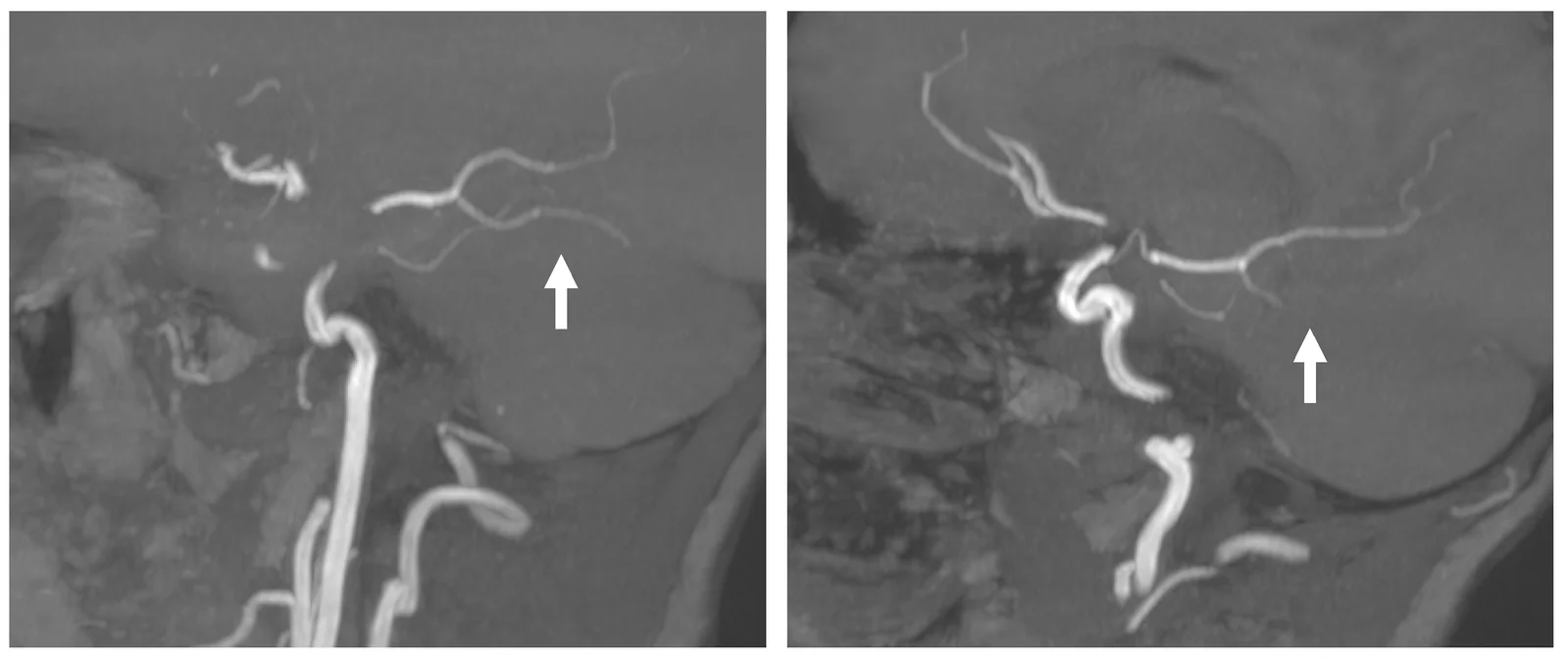
Time Of Flight (TOF) MRA MIP reconstructions of Right and Left PCA arteries from brain MRI study at presentation, demonstrate asymmetry of distal PCA branches (arrows), which are smaller and truncated on the left.

**Figure 4:**
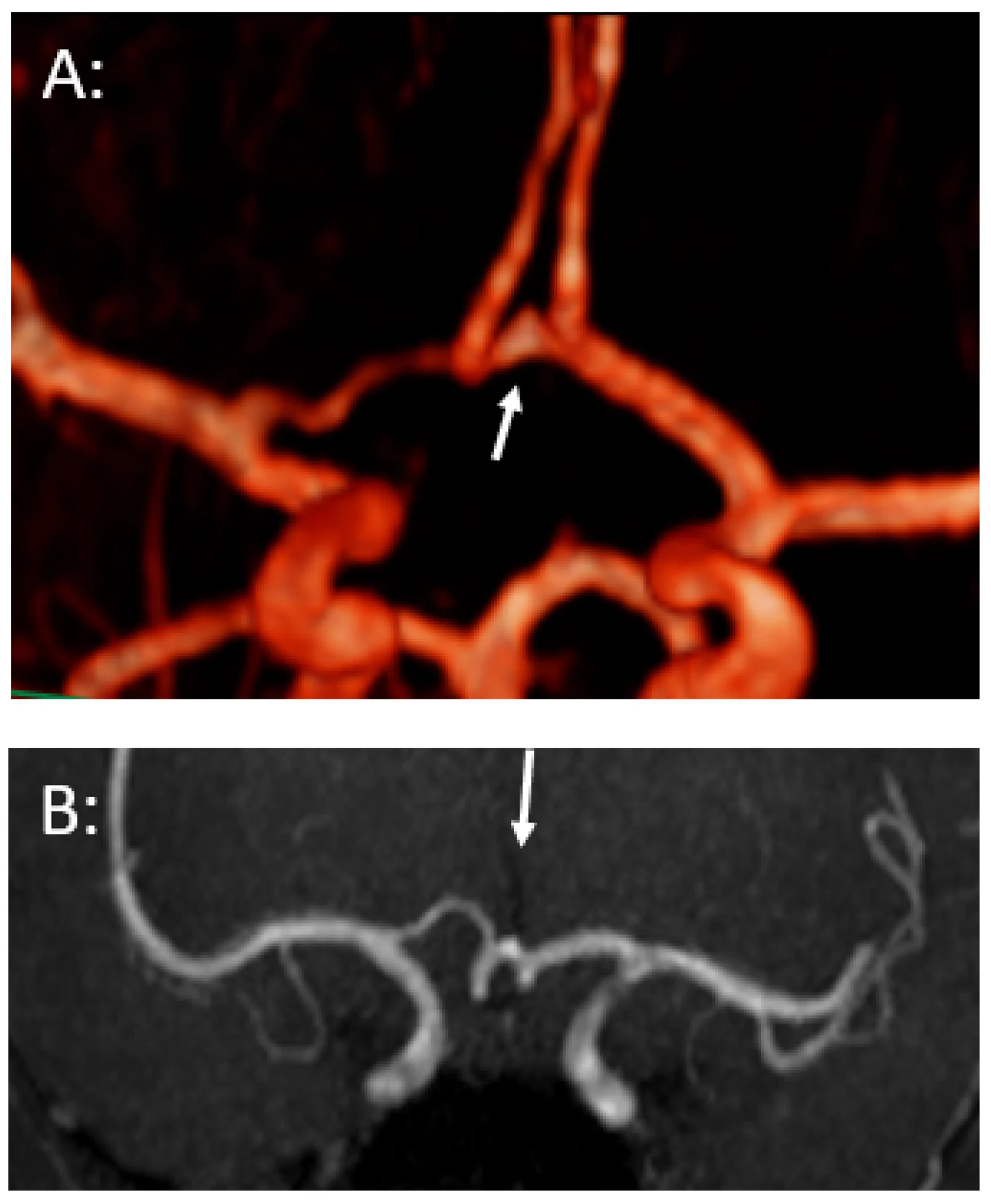
(**A**,**B**): Time Of Flight (TOF) MRA 3D volume rendering image (**A**) and MIP reconstruction image (**B**) of the ACOM demonstrate a small outpouching (arrows) with differential diagnosis including a small saccular aneurysm or a prominent small branch artery infundibulum.

**Table 1:** Cerebrovascular involvement in the *COL4A5*/Alport syndrome spectrum: published reports and the present family.

Reported by	Present Study	Egashira 2024 [12]	Talbot-Stetsko 2023 [11]	Bose 2019 [10]	Wang 2018 [9]	Vaicys 2000 [8]
Gender (Age-years)	Female (16); sister: female (24)	Female (66)	Female (36)	Male (44)	Female (40)	male (14)
Genetic variant	*COL4A5*: c.1871G>A p.Gly624Asp same variant in sister	*COL4A5* c. 1700 G >C p.Gly567Arg	Not reported	Not tested	*COL4A5*: c.C3940>T p.Pro1314Ser and *COL4A6*: c.3538>T p.Pro1180Ser	Not tested
Cerebrovascular manifestation	Proband: microcerebellar hemorrhage. Sister: small saccular aneurysm or branch-artery infundibulum, Acom.	Multiple intracranial stenoses; cerebral infarction	Left vertebral, basilar and both posterior cerebral arteries dissection	Spontaneous SAH due to left vertebral artery dissection extending to the vertebrobasilar junction	FMD; TIAs	Diffuse SAH due to left carotid artery bifurcation aneurysm
Renal involvement	Not specified	Chronic nephritis; proteinuria	End-stage renal disease due to Alport syndrome	ESRD from Alport’s syndrome	Not specified	Renal-biopsy: proven hereditary Alport syndrome
Other clinical features	Proband: segmental neurofibromatosis; recurrent severe migraine attacks. Sister: severe migraine	FH ESRD, hearing loss recurrent transient weakness of the left hand	Not specified	Non-Hodgkin’s lymphoma	Recurrent attacks of vertigo, aphasia hemiplegia	Learning disabilities
Evidence supporting XLAS diagnosis	Variant segregates in the father with XLAS	Two sons with ESRD hearing loss from 20 s; maternal grandmother: CKD and hearing loss.	Clinical reported diagnosis of Alport syndrome	Maternal uncle had ESRD from Alport’s syndrome	Variants segregate in an unaffected mother and daughter	Positive family history of hematuria in the patient’s mother and maternal uncle and grandfather
Familial evidence linking CV events and *COL4A5*	Paternal great grandmother died of a cerebrovascular event in her early 40 s (exact age uncertain). Paternal grandmother: CKD. Paternal grandaunt—stroke at the age of 40, Paternal grandaunt son—died of a cerebrovascular event at the age of 35	SAH in the first son and spinal epidural hematoma in the second son	Not reported	Not reported	Not reported	Not reported

## Data Availability

The data that support the findings of this study are available from the corresponding author upon reasonable request.

## Abbreviations

XLAS-X-linked Alport Syndrome

Acom = Anterior communicating artery; ESRD = end-stage renal disease; SAH = subarachnoid hemorrhage; FMD = fibromuscular dysplasia; CKD = chronic kidney disease; TIA = transient ischemic attack; FH = family history.
